# Molecularly Imprinted Nanozymes for Selective Hydrolysis of Aromatic Carbonates Under Mild Conditions

**DOI:** 10.3390/nano15030169

**Published:** 2025-01-23

**Authors:** Tien Tan Bui, Yan Zhao

**Affiliations:** Department of Chemistry, Iowa State University, Ames, IA 50011-3111, USA

**Keywords:** nanozyme, molecular imprinting, polycarbonate, aromatic carbonate, hydrolysis, micelles, artificial enzymes

## Abstract

Aliphatic polycarbonate (PC) can be readily hydrolyzed by lipase, but bisphenol A-derived PC (i.e., BPA-PC) lacks enzyme catalysts for their efficient hydrolysis due to the high hydrophobicity and rigidity of its polymer backbone. This study aims to develop an artificial nanozyme for the selective hydrolysis of small-molecule aromatic carbonates as model substrates for BPA-PC. The catalyst is prepared through molecular imprinting of cross-linkable micelles in a one-pot reaction using a thiourea template and a zinc-containing functional monomer. The resulting water-soluble nanoparticle resembles a hydrolytic metalloenzyme to bind the appropriately shaped aromatic carbonate substrate in the active site, with the nearby zinc acting as a cofactor to activate a water molecule for the nucleophilic attack on the carbonate. Catalytic hydrolysis is observed at room temperature and pH 7, with a rate acceleration of 1 × 10^6^ for diphenyl carbonate.

## 1. Introduction

The worldwide production of plastics exceeds 500 million metric tons (Mt) per year and is expected to reach 700 Mt by 2030 [1,2]. Less than 30% of waste plastics gets recycled each year, both because of the high costs associated with the collection/processing of the waste and the difficulties in the recycling of some of the polymers. Mechanical recycling of polymers tends to deteriorate the performance of the materials. Chemical recycling has the advantage of producing new polymers from the monomers or oligomers obtained upon depolymerization of the original polymers but is highly challenging except for very limited cases [1].

In principle, step-growth polymers, such as polyesters, polyamides, and polyurethanes, are easier to depolymerize than chain-growth polymers connected by C–C bonds because their acyl-derived backbones can be hydrolyzed by chemical reagents/catalysts or enzyme catalysts. Chemical depolymerization has the advantage of a wide choice of reagents/catalysts and a broad operating window. Nonetheless, the harsh reaction conditions utilized could lead to undesirable side reactions and add significant costs and/or environmental burdens to the process. In contrast, enzymatic degradation could happen under mild conditions in aqueous solutions with minimal side reactions.

Aliphatic PC is hydrolyzed by lipase relatively easily [3]. Good enzymatic catalysts to break down aromatic polycarbonate (PC), however, are practically nonexistent [4]. Aromatic PC, especially those derived from bisphenol A (BPA), contains benzene rings and quaternary carbon atoms in the main chain. Its enzymatic [5], fungal [6], or microbial [7] degradation is sluggish due to the much higher hydrophobicity and rigidity of the polymer backbone.

A method that can potentially bridge the chemical and enzymatic depolymerization of hydrolyzable plastics is to design and synthesize artificial enzymes or nanozymes with hydrolytic activities [8,9]. Ideally, one would like to have catalysts with the efficiency and selectivity of enzymes but a much stronger tolerance for reaction media and temperatures. In recent years, a number of chemical recycling methods of BPA-PC have been reported and reviewed [10,11]. Organic bases such as N,N′-dimethyl-1,2-diaminoethane [12], 1,5,7-Triazabicyclo[4.4.0]-dec-5-ene [13,14], or 1,8-diazabicyclo[5.4.0]undec-7-ene (DBU) [15] at high temperatures are often used for the depolymerization. Researchers have also attempted to make de novo-designed enzymes for BPA-PC hydrolysis [16]. Efficient artificial enzymes for the same purpose, however, have not been reported to the best of our knowledge.

In the past decades, gold nanoparticles have emerged as a versatile platform for designing hydrolytic nanozymes, with the facile assembly of different ligands on their surfaces to create catalytic sites [17,18,19]. It is, however, difficult to create well-defined active sites as in enzymes for specific substrates because a complimentary covalent framework needs to be formed around the substrate, with appropriately distributed binding and catalytic groups.

Our group has been interested in the construction of nanozymes through molecular imprinting of cross-linkable micelles [20,21,22]. As with other molecular imprinting techniques [23,24,25,26,27,28,29,30,31,32], the method relies on the polymerization and cross-linking of functional monomers (FMs) around template molecules to create template-complimentary binding sites within a polymer network. Installation of catalytic groups in the molecularly imprinted binding sites converts the imprinted polymers into artificial enzymes, with some capable hydrolyzing activated esters [23,33,34,35,36]. However, the traditional molecularly imprinted polymers (MIPs) are macroporous polymers, which are microscopic and insoluble. It is challenging to employ an insoluble catalyst to hydrolyze an insoluble (polymer) substrate, due to inaccessibility of the BPA-PC polymer chain to the catalyst. Micellar imprinting, on the other hand, creates water-soluble nanoparticles similar to enzymes, with a hydrophilic exterior and a hydrophobic interior [20,21,22]. Meanwhile, the active site can be tailor-made rationally for different substrates through the imprinting process.

This work represents our initial attempts to build a nanozyme for the hydrolysis of BPA-PC [20]. Although we focus on small-molecule aromatic carbonates as model substrates, the research lays the foundation for using this type of catalyst for the hydrolysis of the polymer. We demonstrate that metal-bound hydroxide can be generated near neutral conditions in the active site of our molecularly imprinted nanozyme to hydrolyze aromatic carbonates efficiently under mild conditions with predesigned substrate selectivity. In addition, the catalysts can be prepared directly from a readily synthesized thiourea template and a zinc-containing functional FM in a one-pot reaction in water within 2 days, underscoring the synthetic efficiency of molecularly imprinted materials.

## 2. Materials and Methods

### 2.1. General Experimental Methods

All reagents and solvents were of ACS-certified grade or higher and used as received from commercial suppliers. Milli-Q water (18.2 MU; MilliporeSigma, Burlington, VT, USA.) was used to prepare buffers and nanoparticles. NMR spectra were recorded on a Bruker DRX-400 (Bruker, Eden Prairie, MN, USA), a Bruker AV III 600 (Bruker, Eden Prairie, MN, USA), or a Varian VXR-400 spectrometer (Varian, Palo Alto, CA, USA). Chemical shifts are reported in ppm relative to residual solvent peaks. Coupling constants are reported in hertz. High-resolution mass spectra (HR-MS) were recorded on an Agilent QTOF 6540 mass spectrometer with a QTOF detector (Agilent, Santa Clara, CA, USA.). Dynamic light scattering (DLS) was recorded at 25 °C on a Malvern Zetasizer Nano ZS instrument (Malvern Panalytical Ltd., Malvern, UK). TEM was analyzed on a 200 kV JEOL 2100 scanning/transmission electron microscope (STEM) (JEOL, Peabody, MA, USA.). UV–vis spectra were recorded on a Cary 50 Bio UV-visible spectrophotometer (Agilent, Santa Clara, CA, USA).

### 2.2. Methods

**Scheme 1:**
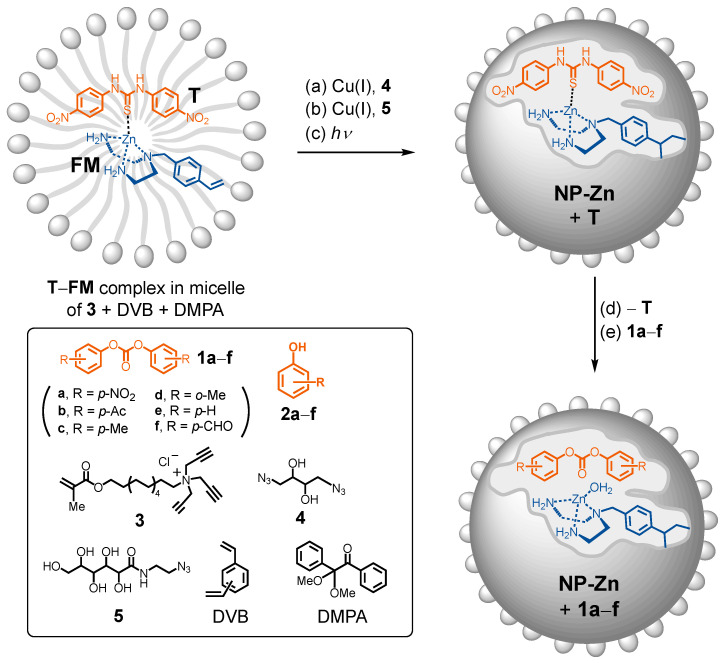
Preparation of nanozyme **NP-Zn** via molecular imprinting of **T**–**FM** complex within the cross-linkable micelle of **3**. The clicked surface ligands **5** are omitted from the drawing for clarity.

The preparation of the zinc-containing nanoparticle (i.e., **NP-Zn**) for diaryl carbonate hydrolysis is shown in Scheme 1. Surfactant **3** [37], diazide surface-cross-linker **4** [37], monoazide surface ligand **5** [37], template **T** [38], and the zinc-containing functional monomer **FM** [39] were synthesized following previously reported procedures.

Compound **1a** has been used as a model substrate for BPA-PC in the de novo design of polycarbonate hydrolases [16], partly because its hydrolysis can be monitored readily by UV–vis spectroscopy. We decided to examine a number of diaryl carbonates (**1b**–**f**) in addition to **1a** (Scheme 1), some of which were used to obtain the Hammett plot for the mechanistic investigation (vide infra). To create a molecularly imprinted site for **1a**, we prepared 1,3-bis(4-nitrophenyl)thiourea as the template (**T**). It is dimensionally similar to **1a**, while its sulfur atom serves as a good ligand for the zinc atom in the tridentate zinc complex **FM**. In this way, the zinc atom would be close to the carbonyl of the bound substrate in the final catalyst. We employed zinc as the catalytic cofactor because it is used to catalyze ester hydrolysis by both natural hydrolases [40,41] and artificial enzymes [42,43,44,45,46,47].

For the preparation, a 1:1 mixture of **T** and **FM** is first solubilized in water by the micelles of **3**. The micelles also contain divinylbenzene (DVB) as a radical cross-linker and 2,2-dimethoxy-2-phenylacetophenone (DMPA) as a photoinitiator. As in other molecularly imprinted nanoparticle catalysts [20,21,22], we first cross-link the surface of the micelles by the Cu-catalyzed cycloaddition between the terminal alkynes on the surfactant headgroups and water-soluble diazide **4** (Scheme 1, step a). We then use the same click reaction to decorate the surface-cross-linked micelles (SCMs) with monoazide **5** (step b). Photoinduced radical polymerization/cross-linking subsequently solidifies the micelle core around the **T**–**FM** complex, covalently attaching the zinc-containing **FM** to the micelle core at the same time (step c). The polyol ligand **5** on the surface of the resulting **NP-Zn** makes it poorly soluble in most organic solvents. The molecularly imprinted nanoparticles can thus be purified by simply precipitation into acetone and solvent washing, with the template removed at the same time (step d). Nonimprinted nanoparticles (NINPs) are also prepared in the absence of the template as the control catalyst.

Our nanoparticles are estimated to contain ~50 surfactants per (cross-linked) micelle. Since a 50:1 surfactant/template ratio is used in the preparation, each nanoparticle, on average, contains a single binding site, a feature previously confirmed by isothermal titration calorimetry (ITC) [37].

#### 2.2.1. General Procedure for the Synthesis of Diaryl Carbonate Substrates **1b**–**f**

An oven-dried 100 mL round-bottom flask equipped with a magnetic stir bar was filled with N_2_ (×3) and charged with the corresponding phenol (4.0 mmol), followed by the addition of anhydrous THF (10 mL) under N_2_. The reaction mixture was cooled to 0 °C before triphosgene (1.0 mmol, 1.0 eq.) and pyridine (5.0 mmol, 5 eq.) were added slowly. The reaction mixture was warmed to room temperature and stirred for 16 h. Upon the completion of the reaction, the resulting suspension was quenched with 1 M HCl (10 mL) and diluted with deionized water (20 mL). The aqueous solution was extracted with ethyl acetate (4 × 30 mL). The combined organic phase was dried over anhydrous Na_2_SO_4_ and concentrated in vacuo. The residue was recrystallized from hot ethanol, followed by washing with a 9:1 mixture of hexane/ethyl acetate to give the desired diaryl carbonates **1b**–**1f**.

**Compound 1b.** An off-white powder (0.38 g, 99%). ^1^H NMR (400 MHz, 298 K, CDCl_3_) δ 8.05 (d, *J* = 8.8 Hz, 4H), 7.40 (d, *J* = 8.8 Hz, 4H), 2.62 (s, 6H). ^13^C{^1^H} NMR (101 MHz, 298 K, CDCl_3_) δ 196.7, 154.3, 150.9, 135.4, 130.3, 121.1, 26.8. HRMS (ESI) *m*/*z*: [M + H]^+^ calcd for C_17_H_15_O_5_ 299.0914; found 299.0915.

**Compound 1c.** A white powder (0.22 g, 92%). ^1^H NMR (400 MHz, 298 K, CDCl_3_) δ 7.20 (d, *J* = 8.2 Hz, 4H), 7.14 (d, *J* = 8.6 Hz, 4H), 2.36 (s, 6H). ^13^C{^1^H} NMR (101 MHz, 298 K, CDCl_3_) δ 152.6, 149.0, 136.1, 130.2, 120.7, 21.0. HRMS (ESI) *m*/*z*: [M + H]^+^ calcd for C_15_H_15_O_3_ 243.1016; found 243.1016.

**Compound 1d.** A white powder (0.24 g, 99%). ^1^H NMR (400 MHz, 298 K, CDCl_3_) δ 7.28–7.23 (m, 4H), 7.22–7.16 (m, 4H), 2.33 (s, 6H). ^13^C{^1^H} NMR (101 MHz, 298 K, CDCl_3_) δ 151.8, 149.7, 131.5, 130.1, 127.3, 126.7, 121.5, 16.1. HRMS (ESI) *m*/*z*: [M + H]^+^ calcd for C_15_H_15_O_3_ 243.1016; found 243.1017.

**Compound 1f.** An off-white powder (0.080 g, 30%). ^1^H NMR (400 MHz, 298 K, CDCl_3_) δ 10.03 (s, 2H), 7.98 (d, *J* = 8.6 Hz, 4H), 7.49 (d, *J* = 8.5 Hz, 4H). ^13^C{^1^H} NMR (101 MHz, 298 K, CDCl_3_) δ 190.8, 155.1, 150.7, 134.7, 131.6, 121.7. HRMS (ESI) *m*/*z*: [M + H]^+^ calcd for C_15_H_11_O_5_ 271.0601; found 271.0601.

#### 2.2.2. Procedure for the Preparation of **NP-Zn** (Scheme 1)

To a micellar solution of compound **3** (8.5 mg, 0.020 mmol) in Milli-Q water (2.0 mL) in a 15 mL glass vial, divinylbenzene (DVB, 1.4 μL, 0.010 mmol), template **T** and **FM** (10 µL of 0.040 M mixture of compound **FM** and **T** in DMSO, 0.00040 mmol), and 2,2-dimethoxy-2-phenylacetophenone (DMPA, 10 μL of a 13 mg/mL solution in DMSO, 0.00050 mmol) were added. The mixture was subjected to ultrasonication for 30 min before compound **4** (4.1 mg, 0.024 mmol), CuCl_2_ (10 μL of a 6.7 mg/mL solution in H_2_O, 0.00050 mmol), and sodium ascorbate (10 μL of a 99 mg/mL solution in H_2_O, 0.0050 mmol) were added. After the reaction mixture was stirred slowly (~60 rpm) at room temperature for 12 h (Scheme 1, step a), compound **5** (16 mg, 0.060 mmol), CuCl_2_ (10 μL of a 6.7 mg/mL solution in H_2_O, 0.00050 mmol), and sodium ascorbate (10 μL of a 99 mg/mL solution in H_2_O, 0.0050 mmol) were added. After being stirred slowly (~90 rpm) for another 6 h at room temperature (step b), the reaction mixture was sealed with a rubber stopper, purged with nitrogen for 15 min, and irradiated in a Rayonet reactor (~254 nm, 300 W/cm^2^) at room temperature for 16 h (step c). ^1^H NMR spectroscopy was used to monitor the progress of the reaction (Figure S1). The reaction mixture was poured into acetone (8.0 mL). The precipitate was collected by centrifugation and washed with a mixture of acetone/water (5.0 mL/1.0 mL) three times to remove the template molecules (step d). The crude product was washed additionally with methanol/acetic acid (5.0 mL/0.10 mL) three times and then with excess methanol (2 × 5.0 mL) and acetone (2 × 5.0 mL). The off-white powder was dried under a high vacuum for 30 min to afford the final **NP-Zn** (14 mg, 70%).

#### 2.2.3. Kinetic Measurement for the Hydrolysis of Diaryl Carbonates

Stock solutions (10.0 mM) of diaryl carbonates in methanol were prepared and used freshly. Stock solutions of the appropriate nanoparticle catalyst (100 µM or 5.0 mg/mL) in a 25 mM HEPES buffer (pH 7.00) were prepared. The concentration of the nanoparticle catalyst is calculated based on a molecular weight of 50 KDa, estimated from dynamic light scattering. For the kinetic experiment, a typical procedure is as follows: An aliquot of the above nanoparticle stock solution (100 µL) was added to the same buffer (1895 µL) in a cuvette. The cuvette was placed in a UV–vis spectrometer and equilibrated at 25.0 °C. After 5 min, a stock solution of carbonate **1b** (5.0 µL) was added to the reaction mixture. The hydrolysis was monitored by the absorbance of 4-acetylphenolate at 324 nm. The rate constant of the hydrolysis was determined by fitting into the pseudo-first-order kinetic model using the equation ln(2 × ε × l × [**1b**] − A_t,324nm_) = ln(2 × ε × l × [**1b**]) − k × t, in which ε is the extinction coefficient, A is absorbance, l is path length of light, t is time in seconds, and k is rate constant.

The hydrolysis of *para*-substituted diaryl carbonates was performed at 25 °C using the typical procedure as described above in 25 mM HEPES buffer pH = 7.00.

## 3. Results and Discussion

Scheme 1 illustrates the preparation of the zinc-containing nanoparticle catalyst (i.e., **NP-Zn**). The surface- and core-cross-linking of the micelles were monitored by ^1^H NMR spectroscopy (Figure S1). The resulting **NP-Zn** was characterized by dynamic light scattering (DLS) and transmission electron microscopy (TEM) for its particle size (Figures S2–S5).

We initially used **1a** as the substrate to evaluate the hydrolytic activity of **NP-Zn** but found that the compound hydrolyzes too quickly under our experimental conditions (pH 7 at 25 °C). We then employed a less reactive substrate (**1b**) to evaluate the performance of our catalyst.

**Figure 1:**
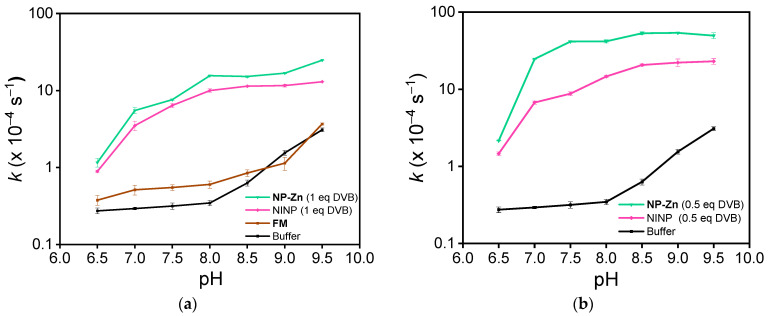
(**a**) Dependence of the pseudo-first-order rate constant for the hydrolysis of **1b** on the solution pH at 25 °C. The nanoparticles were prepared using 1.0 equiv of DVB; (**b**) Dependence of the pseudo-first-order rate constant for the hydrolysis of **1b** on the solution pH at 25 °C. The nanoparticles were prepared using 0.5 equiv of DVB. [**1b**] = 25 μM. [NINP] = 5 μM.

Figure 1a shows the reaction rates for the hydrolysis of **1b** in aqueous buffer over pH 6.5–9.5. The background hydrolysis in the buffer is slow below pH 8 and increases steadily above pH 8. This is consistent with base-promoted hydrolysis of the carbonate with a higher concentration of hydroxide under more basic conditions [48]. The presence of the (unpolymerized) zinc-containing **FM** in the solution makes very little difference in the hydrolysis rate, while both NINP and **NP-Zn** speed up the hydrolysis strongly. The largest rate acceleration by the nanoparticles over the background hydrolysis occurs over pH 6.5–7.5.

Cationic micelles are known to help deprotonation of water near the micelle surface by their electric potentials. The surface of the micelles of cetyltrimethylammonium bromide (CTAB), for example, has a local pH of 9.5 when the bulk solution is neutral [49]. The effect of the electric potential is stronger when the micelle is cross-linked [50]. Thus, the higher reactivity of **1b** in the presence of cross-linked cationic micelles (NINP) is not surprising. A more important question is whether **NP-Zn** is able to catalyze the hydrolysis of diaryl carbonates, as hypothesized in Scheme 1.

The molecularly imprinted catalyst (**NP-Zn**) is only slightly more active as a catalyst than the control NINP in Figure 1a. These nanoparticles were prepared with 1 equivalent of DVB to surfactant **3**. This amount of DVB is the maximum that can be solubilized by **3** in water and has been shown to be critical to the integrity of the imprinted site [37]. Given that the substrate is large and hydrophobic, its entrance into the active site and the exit of the hydrolyzed product (i.e., the corresponding phenol **2b**) might be hindered by the rigid polymer network. To increase the flexibility of the cross-linked micelle, we prepared **NP-Zn** and NINP with half of the amount of DVB (0.5 equiv to **3**). To our delight, the imprinted catalyst shows significant improvements in its activity (compare the green lines in Figure 1a,b), while the corresponding NINP displays little change over that prepared with 1 equiv DVB (compare the magenta lines in Figure 1a,b).

**Figure 2:**
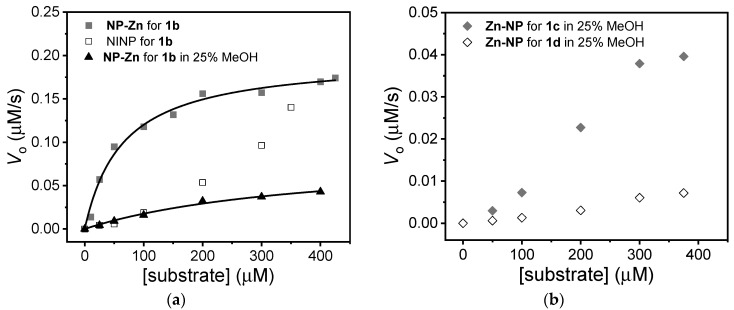
(**a**) Dependence of initial velocity (*V*_0_) on the substrate concentration for the hydrolysis of **1b** by **NP-Zn** and NINP in a 25 mM HEPES buffer (pH 7.0) at 25 °C and fitting of the **NP-Zn** data to the Michaelis-Menten model. [**NP-Zn**] = [NINP] = 5.0 μM. (**b**) Dependence *V*_0_ on the substrate concentration for the hydrolysis of **1c** and **1d** by **NP-Zn** in a 25 mM HEPES buffer (pH 7.0) containing 25 vol % methanol at 25 °C. [**NP-Zn**] = 5.0 μM. The nanoparticles were prepared using 0.5 equiv of DVB.

Figure 2a compares the initial velocity (*V*_0_) for the catalytic hydrolysis of **1b** by **NP-Zn** and NINP prepared with 0.5 equiv DVB. Importantly, the hydrolysis by **NP-Zn** exhibits a saturation behavior, typical of enzymes (solid square data points). The data fits well to the Michaelis–Menten model, affording a *k*_cat_ of (39.5 ± 0.6) × 10^−3^ s^−1^ and a *K*_M_ of 64.4 ± 3.0 μM (Table 1, entry 1). In contrast, no saturation in catalysis is observed for the hydrolysis by NINP (empty square data points), suggesting that a different catalytic mechanism is involved. The reaction by NINP displays a slower and a faster region, and more efficient catalysis is achieved only at high substrate concentrations (>300 μM). Below 200 μM of **1b**, **NP-Zn** is clearly a better catalyst for the hydrolysis than NINP.

A parameter frequently used to evaluate the effectiveness of molecular imprinting is the imprinting factor, frequently determined by the ratio in the binding of the imprinted polymer for the template over that of the nonimprinted polymer. Although the insolubility of the template **T** in water makes direct determination of the binding constants challenging, we could use the *K*_M_ values of the imprinted and nonimprinted nanoparticles to estimate the imprinting factor. Although hydrolysis of **1b** by NINP does not give saturation behavior, the data does fit to the Lineweaver–Burk plot (Figure S6) to give a *k*_cat_ of (7.2 ± 1.3) × 10^−3^ s^−1^ and a *K*_M_ of 350 ± 15 μM (Table 1, entry 2). Thus, an imprinting factor of 5.5 can be obtained if the *K*_M_ values of **NP-Zn** and NINP are used. The imprinting factor based on the catalytic efficiency (*k*_cat_/*K*_M_) is significantly higher, ca. 30.

If the catalytic hydrolysis occurs in the active site of **NP-Zn**, as portrayed in Scheme 1, substrate selectivity should also be expected. To test this hypothesis, we studied the hydrolysis of **1c** and **1d**, the diphenyl carbonate derivative, with a *para* and an *ortho* methyl group on the phenyl rings. Because the template (**T**) has a *para* nitro group on the phenyl rings, the resulting active site should be able to accommodate **1c** but exclude **1d** by the mismatched geometry. Indeed, the matched *para*-methyl substrate **1c** is reasonably active in its catalytic hydrolysis by **NP-Zn** (Figure 2b, solid diamond data points), whereas the mismatched **1d** with the *ortho*-methyl groups displays a negligible reactivity (Figure 2b, empty diamond data points). It should be mentioned that **1c** and **1d**, due to their higher hydrophobicity, are insoluble in water at high concentrations, and 25 vol % methanol was added to keep the reaction mixture homogeneous for the Michaelis–Menten experiments (Figure S7).

In our current design, the template (**T**) resembles the desired substrates (e.g., **1b** and **1c**) so that the imprinted active site will have an affinity for the substrate (akin to the *K*_M_ value in an enzymatic reaction). The affinity not only increases the effective concentration of the substrate near the catalytic groups but also positions the reactive group (i.e., the carbonyl) near the catalytic cofactor (i.e., the zinc atom). For a “lock-and-key” system, the shape of the active site is key to substrate selectivity. For the same reason, the current catalyst is not expected to hydrolyze a polymeric substrate such as BPA-PC because its long hydrophobic backbone cannot be accommodated by the active site of **NP-Zn**.

Table 1 summarizes the Michaelis–Menten parameters for the catalytic hydrolysis of several carbonate substrates. The *k*_cat_ value is the highest for **1b**, the compound with the best leaving group (entry 1). We also studied the hydrolysis of this compound in 25% methanol (entry 3) so that it can be compared directly with **1c** in the same solvent (entry 4). Compounds **1b** and **1c**, having a reasonably sized *para*-substituent on the phenyl rings, are both expected to fit within the active site of **NP-Zn** due to their similarity to the template in size and shape (Scheme 1). In 25% methanol, **1b** has a 3.2× turnover number and ~30% stronger binding than **1c**. In the end, the catalytic efficiency (*k*_cat_/*K*_M_) is four times higher for **1b** than for **1c**. When **1b** and **1e** are compared, the catalytic efficiency for the two substrates differs by a factor of 22, with positive contribution from both *k*_cat_ (a factor of 3.3) and *K*_M_ (a factor of 6.6). In other words, the dominant factor is the binding affinity for the better substrate.

If we compare the hydrolysis of **1b** in water and 25% methanol, the *k*_cat_ value decreases by 2.3 times, but the binding weakens by 6 times (Table 1, entries 1 and 3). The result confirms the hydrophobically driven binding of the substrate, as the addition of an organic solvent (methanol) to the reaction medium is expected to significantly reduce the driving force for the substrate to enter the active site. The hydrophobically driven binding is also supported by the weak binding of **1e** (entry 4). With only hydrogen on the *para* positions, **1e** could only fill part of the active site. The situation is unfavorable whether the rest of the active site is left empty or filled with water molecules.

The literature-reported rate constant (*k*_uncat_) for the uncatalyzed hydrolysis of diphenyl carbonate **1e** in water is 1.2 × 10^−8^ s^−1^ [33]. Thus, **NP-Zn** is able to accelerate the hydrolysis by ca. 1 × 10^6^ at pH 7 and room temperature. A de novo designed thermostable polycarbonate hydrolase has an activity of 18.2 μM/(min·mg) for **1a** at 25 °C in pH 8 buffer [16]. As mentioned above, this particular substrate reacts too fast under our experimental conditions. Nonetheless, **NP-Zn** is able to turn over the less reactive **1b** with an activity of 23.6 μM/(min·mg) at 25 °C in pH 7 buffer. Thus, the activity is about 30% higher for a less reactive substrate under a less basic solution, underscoring the efficiency of our artificial nanozyme.

**Figure 3:**
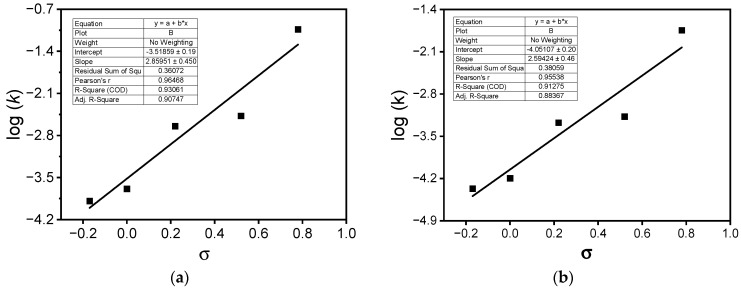
(**a**) Hammett σ–ρ correlation in the hydrolysis of *para*-substituted diaryl carbonates catalyzed by (a) **NP-Zn** and (**b**) NINP. Reaction rate constants were measured in 25 mM HEPES buffer (pH 7.0) at 25 °C. [carbonates] = 25 μM. [**NP-Zn**] = [NINP] = 5 μM. σ values p-NO_2_, 0.78; p-CH_3_CO, 0.52; p-CHO, 0.22; p-H, 0.00; p-CH_3_, -0.17. The nanoparticles were prepared using 0.5 equiv of DVB.

Linear free energy relationships can provide significant mechanistic insights into both solution-based and enzyme-catalyzed reactions [51,52]. When **NP-Zn** and NINP are used to hydrolyze a series of *para*-substituted phenyl carbonates, linear relationships are observed in both cases, with the reaction constant (ρ) of 2.86 (Figure 3a) and 2.59 (Figure 3b), respectively. These numbers are similar to those reported for the hydrolysis of aromatic carbonates by hydroxide, i.e., ρ = 2.7–3.2 [48]. As discussed earlier, the hydroxide ions in the NINP catalyst are most likely those near the surface of the micelles, generated with the help of the electric potential of the cationic micelles [49,50]. For **NP-Zn**, the zinc cofactor is proposed to use a metal-bound hydroxide for the nucleophilic attack on the carbonate substrate. Essentially, the Lewis acidic zinc helps a metal-bound water molecule to deprotonate more easily to generate a stronger nucleophile (i.e., hydroxide) in the active site. The p*K*_a_ of [Zn(OH_2_)_6_]^2+^ is 9.0 [53], but the many cationic charges on the cross-linked micelles can help the deprotonation of the water, similar to what happens in many enzymes [54]. Consistent with the picture, the hydrolysis of **1b** by **NP-Zn** is less sensitive to the solution pH than the NINP control over pH 7.0–9.5 in Figure 1b. When external hydroxide ions are involved in the hydrolysis, the reaction is most sensitive to the solution pH (at least above pH 8.0), as shown by the reaction in the buffer in Figure 1b.

Further support for the hydroxide-based hydrolysis in **NP-Zn** comes from the kinetic solvent isotope effects, which are often used to distinguish a nucleophilic mechanism by hydroxide from a general base catalysis [55,56,57,58]. The former does not involve O–H bond cleavage in the rate-determining step, affording a kinetic solvent isotope effect of *k*_H2O_/*k*_D2O_ ≈ 1. A general base mechanism, characterized by a cleavage of the water O–H bond in the rate-determining step, often exhibits a primary solvent isotope effect, with *k*_H2O_/*k*_D2O_ = 2–3. In our hands, a *k*_H2O_/*k*_D2O_ value of 1.04 was observed for the hydrolysis of **1b** by NP-Zn at pH 7 (Table S1), consistent with the postulation that a nucleophilic attack by hydroxide is responsible for the catalysis. In this experiment, the pD value was determined by adding 0.4 to the pH meter reading since water and D_2_O have different dissociation constants [56].

## 4. Conclusions

Molecular imprinting in cross-linked micelles affords a facile method to prepare hydrolytic nanozymes for aromatic carbonates. An aromatic thiourea template resembles the substrate in size/shape and uses its sulfur atom to coordinate to a zinc-containing functional monomer. Double cross-linking of cross-linkable micelles containing the above template–FM complex produces water-soluble nanoparticles as enzyme mimics in a straightforward fashion.

Our study reveals that the zinc cofactor is able to accelerate the hydrolysis of aromatic carbonates substantially, with a rate acceleration of 1 × 10^6^ for diphenyl carbonate, a nonideal substrate. The involvement of the molecularly imprinted site for the catalysis is supported by the strong reactivity of the *para*-substituted substrate **1c** and the low reactivity of the mismatched isomeric **1d**. Mechanistic studies support that the most likely mechanism for the hydrolysis comes from local hydroxide generated by the active site zinc cofactor. For more reactive carbonates (e.g., **1b**), the cationic micelle can also generate hydroxide ions near the micelle surface by its electric potential, which contribute to the hydrolysis at high substrate concentrations. These doubly cross-linked micellar catalysts have extraordinary abilities to tolerate high temperatures [22], organic solvents [59], and adverse pH conditions [60]. As mentioned earlier, the current catalyst **NP-Zn** is unable to accommodate (a segment of) the BPA-PC chain and is only designed to hydrolyze the small molecule substrates. The designability of the molecularly imprinted catalysts, however, should enable a catalyst for hydrolyzing the polymer using more elaborate template molecules. This work has begun in our laboratory and will be reported in due course.

## Figures and Tables

**Table 1:** Michaelis–Menten parameters for the catalytic hydrolysis of aromatic carbonates by nanoparticle catalysts.

Entry	Substrate	Catalyst	*k*_cat_ (×10^−3^ s^−1^)	*K*_M_ (μM)	*k*_cat_/*K*_M_ (M^−1^s^−1^)	*k*_cat_/*k*_uncat_
1	**1b** ^a^	**NP-Zn**	39.5 ± 0.6	64 ± 3	610	-
2	**1b** ^a^	NINP	7.2 ± 1.3	350 ± 15	20	-
3	**1b** ^b^	**NP-Zn**	17.2 ± 0.2	387 ± 19	44	-
4	**1c** ^b^	**NP-Zn**	5.4 ± 0.3	496 ± 3	11	-
5	**1e** ^a,c^	**NP-Zn**	11.9 ± 0.3	425 ± 1	28	1.0 × 10^6^

^a^ Hydrolysis was performed in a 25 mm HEPES buffer (pH 7.0) at 25 °C. [catalyst] = 5.0 μM. ^b^ Hydrolysis was performed in a 25 mm HEPES buffer (pH 7.0) containing 25 vol % methanol at 25 °C. [**NP-Zn**] = 5.0 μM. ^c^ The uncatalyzed rate constant (*k*_uncat_) for the hydrolysis of diphenyl carbonate **1e** is 1.2 × 10^−8^ s^−1^ [33].

## Data Availability

The original contributions presented in this study are included in the article/Supplementary Materials. Further inquiries can be directed to the corresponding author.

## Supplementary Materials

The following supporting information can be downloaded at https://www.mdpi.com/article/10.3390/nano15030169/s1, Figure S1: Monitoring of the surface- and the core-cross-linking of the micelles of **3** during the molecular imprinting by ^1^H NMR spectroscopy; Figure S2: Distribution of the hydrodynamic diameters of the nanoparticles in water as determined by DLS for alkynyl-SCM (surface-cross-linked micelle); Figure S3: Distribution of the hydrodynamic diameters of the nanoparticles in water as determined by DLS for surface functionalized SCM (surface-cross-linked micelle); Figure S4: Distribution of the hydrodynamic diameters of the nanoparticles in water as determined by DLS for **NP-Zn**; Figure S5: Bright-field TEM image of typical molecularly imprinted nanoparticles; Figure S6: Lineweaver-Burk plot of the hydrolysis of **1b** by NINP in a 25 mM HEPES buffer (pH 7.0) at 25 °C; Figure S7: Lineweaver-Burk plot of the hydrolysis of **1c** by **NP-Zn** in a 25 mm HEPES buffer (pH 7.0) containing 25 vol % methanol; Table S1: Pseudo-first-order rate constants and solvent kinetic isotope effects of the hydrolysis of compound **1b** catalyzed by **NP-Zn** at 25 °C.
