# Supplementary material for: Molecularly Imprinted Nanozymes for Selective Hydrolysis of Aromatic Carbonates Under Mild Conditions
Source: Nanomaterials (Basel). 2025 Jan 23;15(3):169. doi: 10.3390/nano15030169 (PMC11820003; doi:10.3390/nano15030169)
Supplement: Supplementary file 1 [file nanomaterials-15-00169-s001.zip › nanomaterials-3392625-supplementary.pdf]

Supplementary Materials

Molecularly Imprinted Nanozymes for Selective Hydrolysis of Aromatic Carbonates under Mild Conditions

Tien Tan Bui and Yan Zhao*

Department of Chemistry, Iowa State University, Ames, Iowa 50011-3111, USA

**zhaoy@iastate.edu*

Table of Contents

Reagents and Stock Solutions S2
Characterization of MINPs S3
Figure S1. .... S3
Dynamic Light Scattering S3
Figure S2. .... S4
Figure S3. .... S4
Figure S4. .... S5
Figure S5. .... S5
Figure S6. .... S6
Figure S7. .... S6
^{1}H and $^{13}C\{^{1}H\}$ NMR spectra of key compounds S7

Reagents and Stock Solutions

1-(4-hydroxyphenyl)ethan-1-one (phenol for preparation of compound **1b**)
p-cresol (phenol for preparation of compound **1c**)
o-cresol (phenol for preparation of compound **1d**)
4-hydroxybenzaldehyde (phenol for preparation of compound **1f**)
Triphosgene
Pyridine
Tetrahydrofuran (THF)
Ethyl acetate (EtOAc)
Hexanes
Ethanol
Methanol
Acetone
Acetic acid
Aqueous hydrochloric acid (HCl, 1M)
Sodium sulfate (anhydrous)
bis(4-nitrophenyl) carbonate (compound **1a**)
bis(4-acetylphenyl) carbonate (compound **1b**)
di-p-tolyl carbonate (compound **1c**)
di-o-tolyl carbonate (compound **1d**)
diphenyl carbonate (compound **1e**)
bis(4-formylphenyl) carbonate (compound **1f**)
12-(methacryloyloxy)-*N*, *N*, *N*-tri(prop-2-yn-1-yl)dodecan-1-aminium chloride (compound **3**, cationic surfactant containing alkynes)
1,4-diazidobutane-2,3-diol (compound **4**, surface cross-linking azide)
N-(2-azidoethyl)-2,3,4,5,6-pentahydroxyhexanamide(compound **5**, surface-decorating azide)
1,3-bis(4-nitrophenyl)thiourea (compound **T**)
N^1-(2-aminoethyl)-N^1-(4-vinylbenzyl)ethane-1,2-diamine, zinc (II) perchlorate trihydrate (compound **FM**)
Divinylbenzene (DVB)
2,2-dimethoxy-2-phenylacetophenone (DMPA)
Copper (II) chloride ($CuCl_2$)
Sodium ascorbate
Nanoparticle imprinted with compound **T** and compound **FM** (NP-Zn)
Nonimprinted nanoparticles (NINPs)
2-(*N*-morpholino)ethanesulfonic acid (MES, for buffer preparation with pH = 6.5)
4-(2-Hydroxyethyl)piperazine-1-ethane-sulfonic acid (HEPES, for buffer preparation with pH range from 7.0 – 8.5)
N-cyclohexyl-2-aminoethanesulfonic acid (CHES, for buffer preparation with pH range from 9.0 – 9.5)

Characterization of MINPs

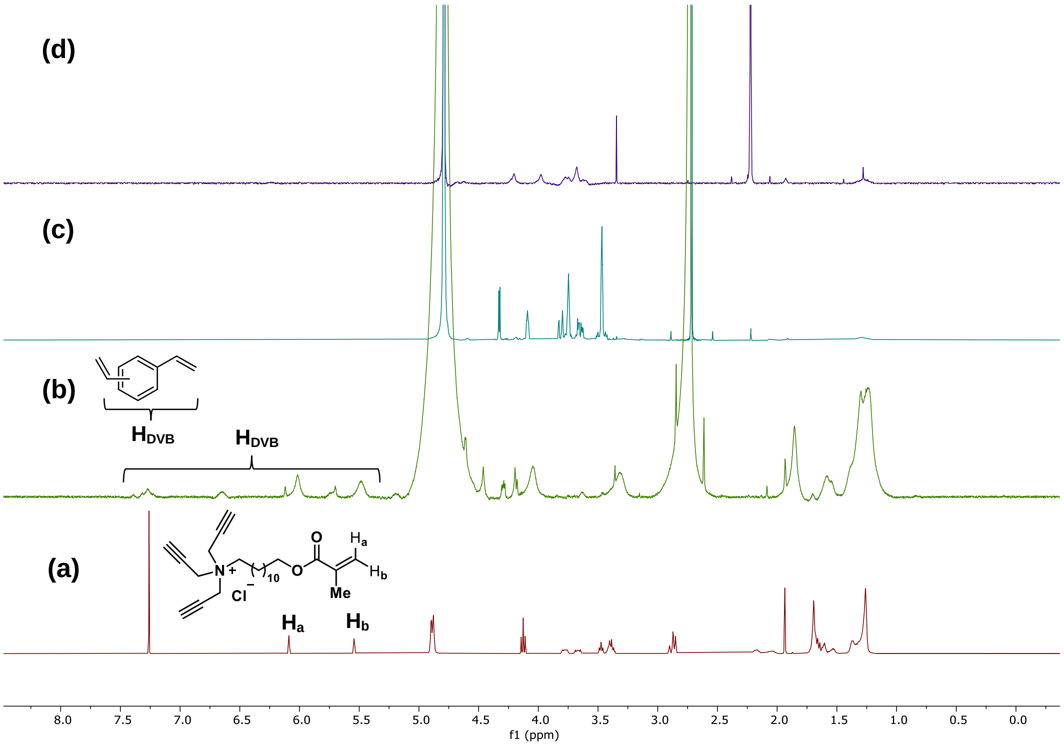

Figure S1. Monitoring of the surface- and the core-cross-linking of the micelles of **3** during the molecular imprinting by ^{1}H NMR spectroscopy. ^{1}H NMR spectra of (a) surfactant **3** in $CDCl_3$, (b) typical alkynyl surface cross-linked micelle in D_2O, (c) typical surface-functionalized micelle in D_2O, (d) typical **NP-Zn** after washing and re-dissolving in D_2O.

Dynamic Light Scattering

The particle size of MINP was determined on a Malvern Zetasizer Nano ZS using the Zetasizer software according to the Stokes-Einstein equation (1). The volume of a spherical nanoparticle (V_{D_h}) was calculated from equation (2). Assuming a density of 1.37 g/cm^3 (the density of protein), the molecular weight of the particle can be calculated using equation (3).[1] A nanoparticle with a hydrodynamic diameter of 4.87 nm has a calculated molecular weight of 50 kDa, which was used in making MINP solution for ITC titration.

---

[1] Erickson, H. P. Size and Shape of Protein Molecules at the Nanometer Level Determined by Sedimentation, Gel Filtration, and Electron Microscopy. *Biological Procedures Online* **2009**, *11*, 32.

$$D_h = \frac{k_B T}{6\pi\eta D_t} \quad (1)$$

in which D_h is the hydrodynamic diameter, D_t the translational diffusion coefficient measured by dynamic light scattering, T the temperature, k_B the Boltzmann's constant, and η is dynamic viscosity of water (0.890 cP at 298 K).

$$V_{D_h} = \frac{4\pi}{3}\left(\frac{D_h}{2}\right)^3 \quad (2)$$

$$\text{Mw in dalton} = \left(\frac{D_h}{0.132}\right)^3 \quad (3)$$

in which D_h is the hydrodynamic diameter in nm.

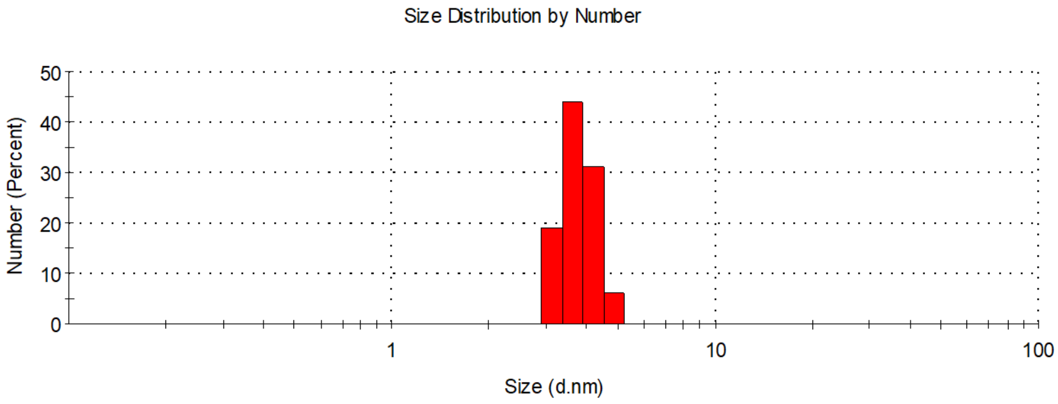

Figure S2. Distribution of the hydrodynamic diameters of the nanoparticles in water as determined by DLS for alkynyl-SCM (surface-cross-linked micelle). D = 3.86 ± 0.28 nm.

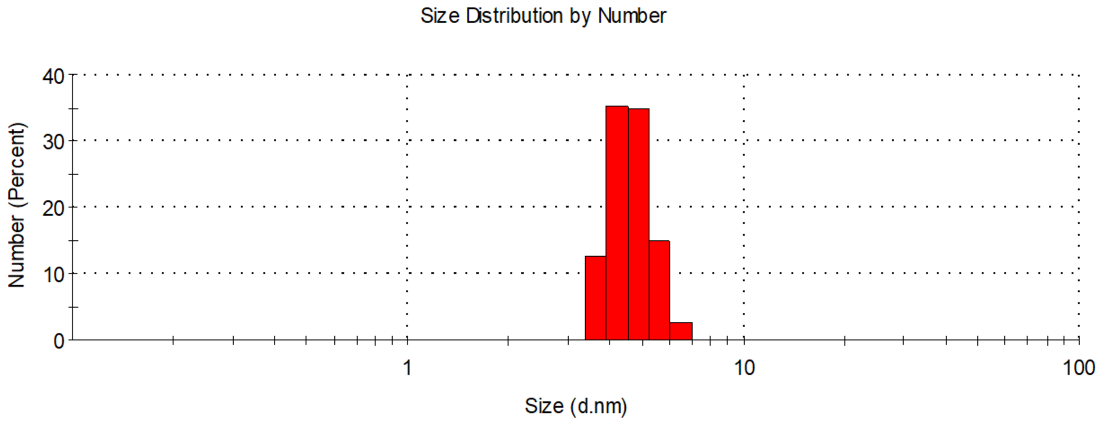

Figure S3. Distribution of the hydrodynamic diameters of the nanoparticles in water as determined by DLS for surface functionalized SCM (surface-cross-linked micelle). D = 4.90 ± 0.52 nm.

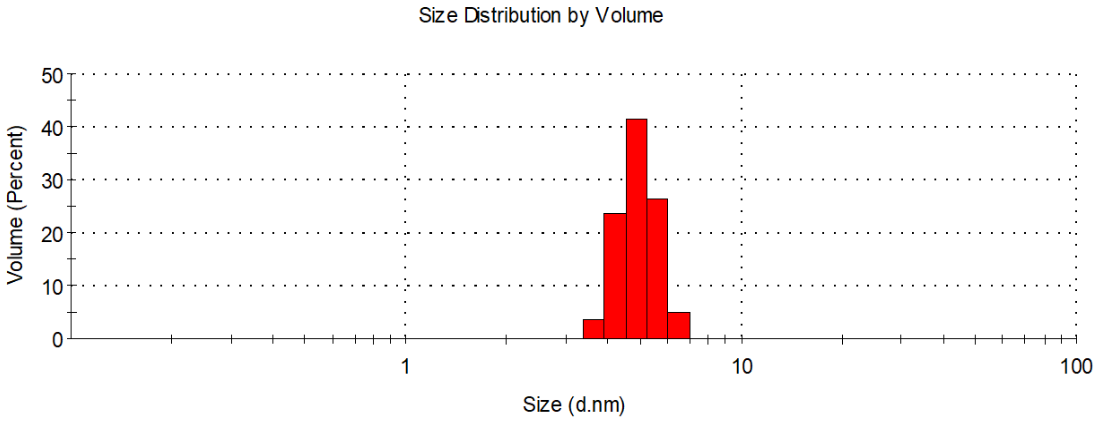

Figure S4. Distribution of the hydrodynamic diameters of the nanoparticles in water as determined by DLS for **NP-Zn**. D = 5.01 ± 0.42 nm.

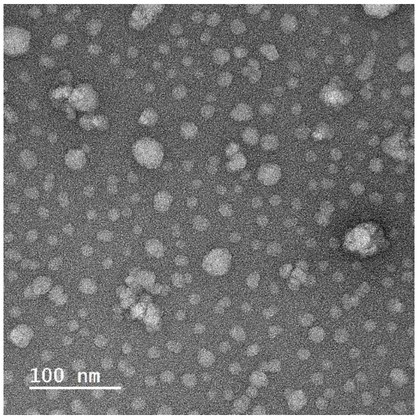

Figure S5. Bright-field TEM image of typical molecularly imprinted nanoparticles (scale bar = 100 nm). For the TEM imaging, 0.1 mg of MINP was dissolved in 1 mL of Millipore water, and the solution was ultra-sonicated for 10 min. A microsyringe was used to load one small drop (~1 μL) of the above solution onto a TEM copper grid covered with carbon film. The sample was left to form a thin layer, and then one small drop (~1 μL) of 2% uranyl acetate solution was loaded on the grid for the negative staining. The sample was left to dry and analyzed on a 200kV JEOL 2100 scanning/transmission electron microscope (STEM).

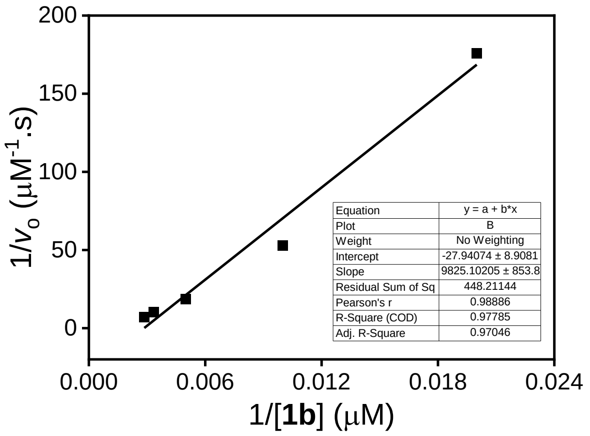

Figure S6. Lineweaver-Burk plot of the hydrolysis of **1b** by NINP in a 25 mM HEPES buffer (pH 7.0) at 25 °C. [NINP] = 5 μM.

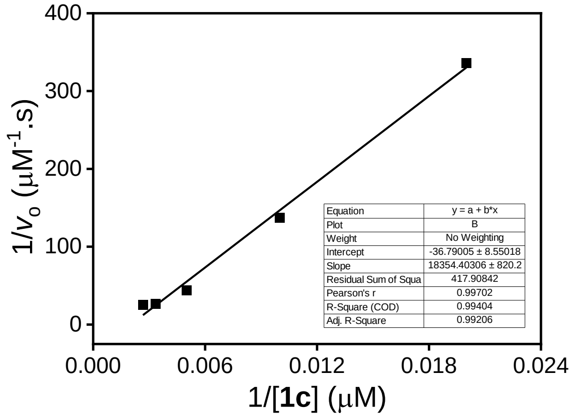

Figure S7. Lineweaver-Burk plot of the hydrolysis of **1c** by **NP-Zn** in a 25 mM HEPES buffer (pH 7.0) containing 25 vol % methanol at 25 °C. [**NP-Zn**] = 5 μM.

Table S1. Pseudo-first-order rate constants and solvent kinetic isotope effects of the hydrolysis of compound **1b** catalyzed by **NP-Zn** at 25 °C.[a]

Entry	pH or pD	System	k_{H2O} or k_{D2O} ($10^{-4}.s^{-1}$)	k_{H2O} / k_{D2O}
1	7.00	H_2O	32.9 ± 3.0	1.04
2	7.00	D_2O	31.7 ± 1.0	

[a]Reaction rate constants were measured by monitoring the formation of 4-acetylphenolate at 324 nm. [**1b**] = 25 μM, [**NP-Zn**] = 5.0 μM. The pD values were determined by adding 0.40 to the pH meter reading.

^{1}H and ^{13}C{^{1}H} NMR spectra of key compounds

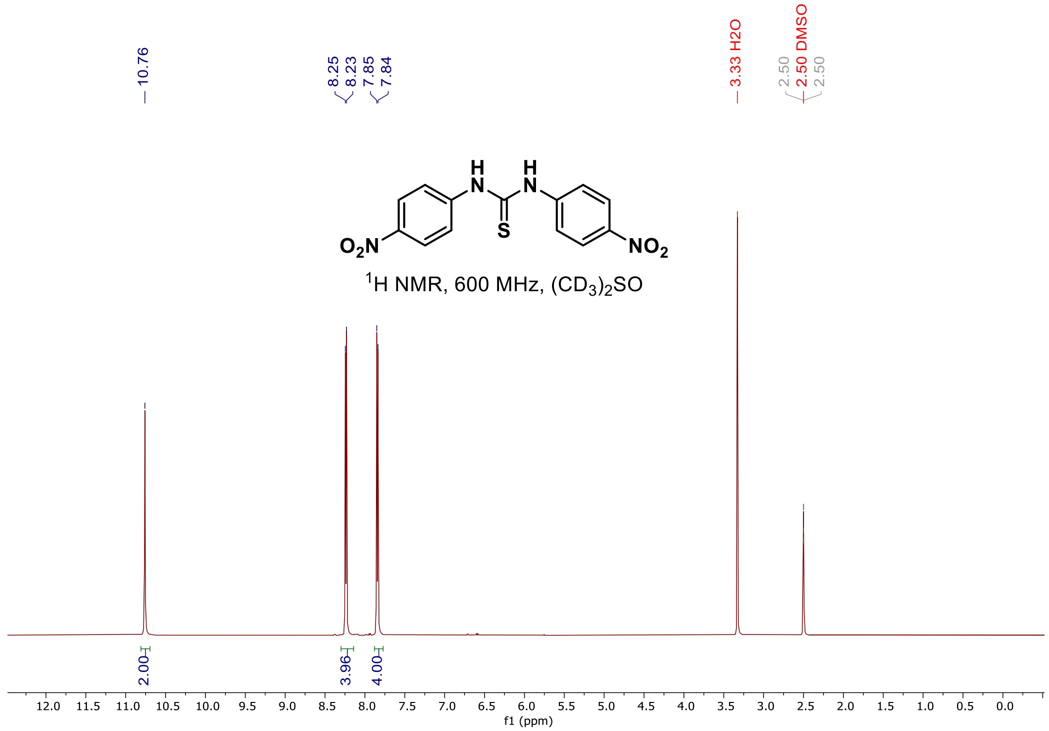

^{1}H NMR spectrum of compound **T**

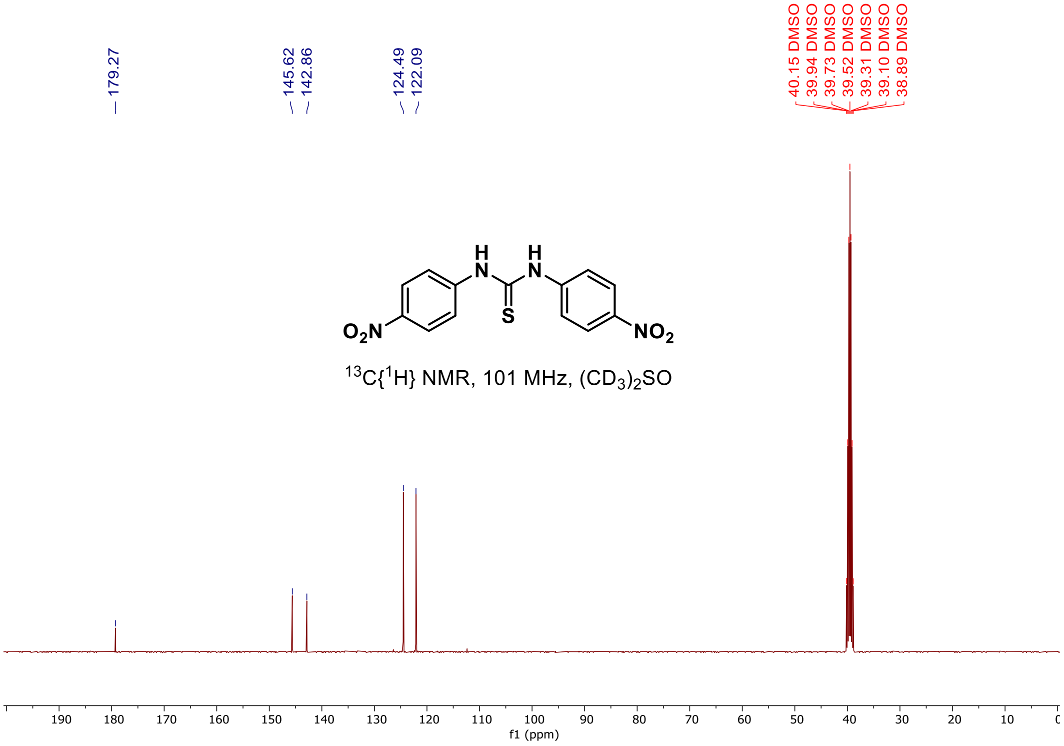

^{13}C NMR spectrum of compound **T**

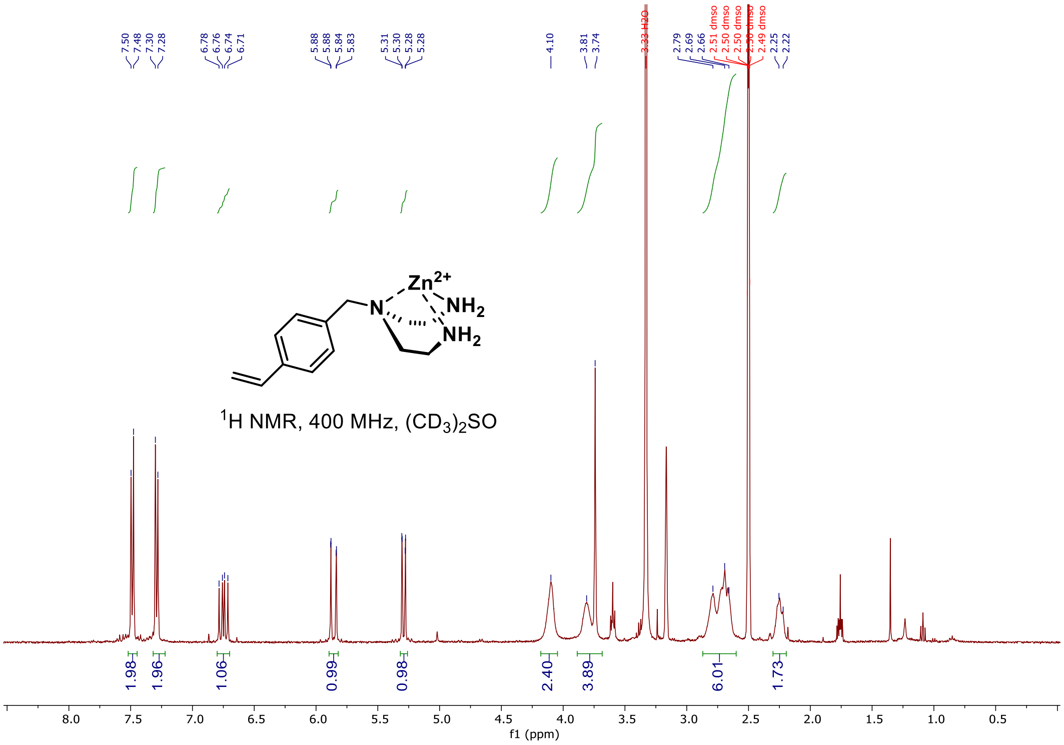

^{1}H NMR spectrum of compound **FM**

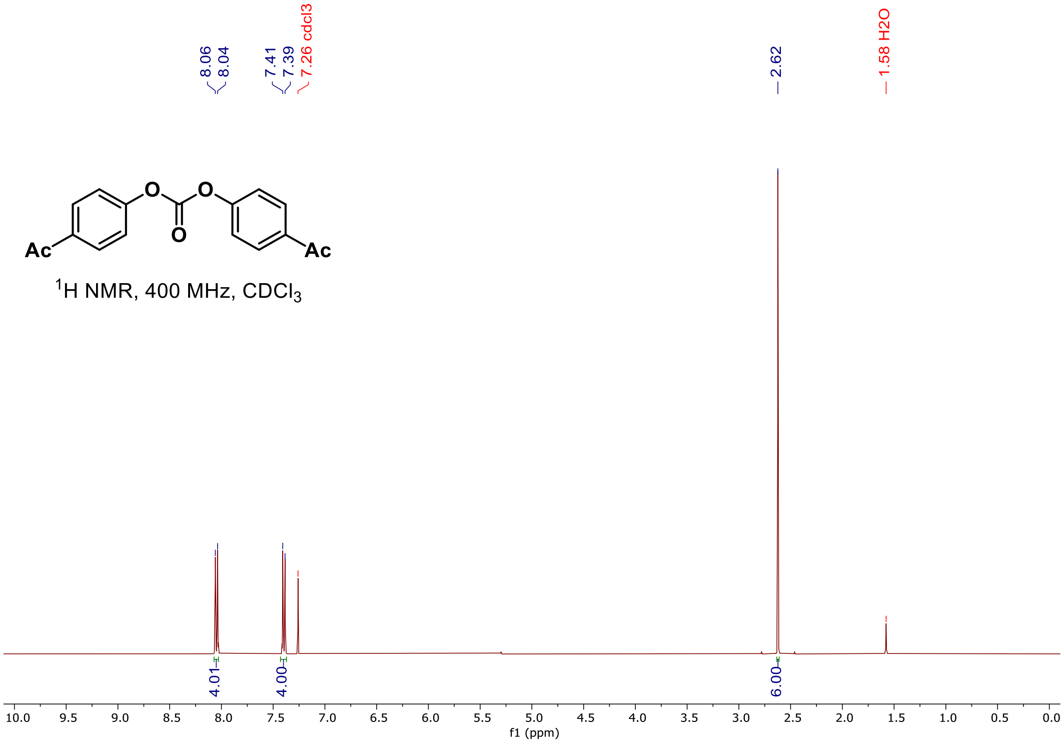

^{1}H NMR spectrum of compound **1b**

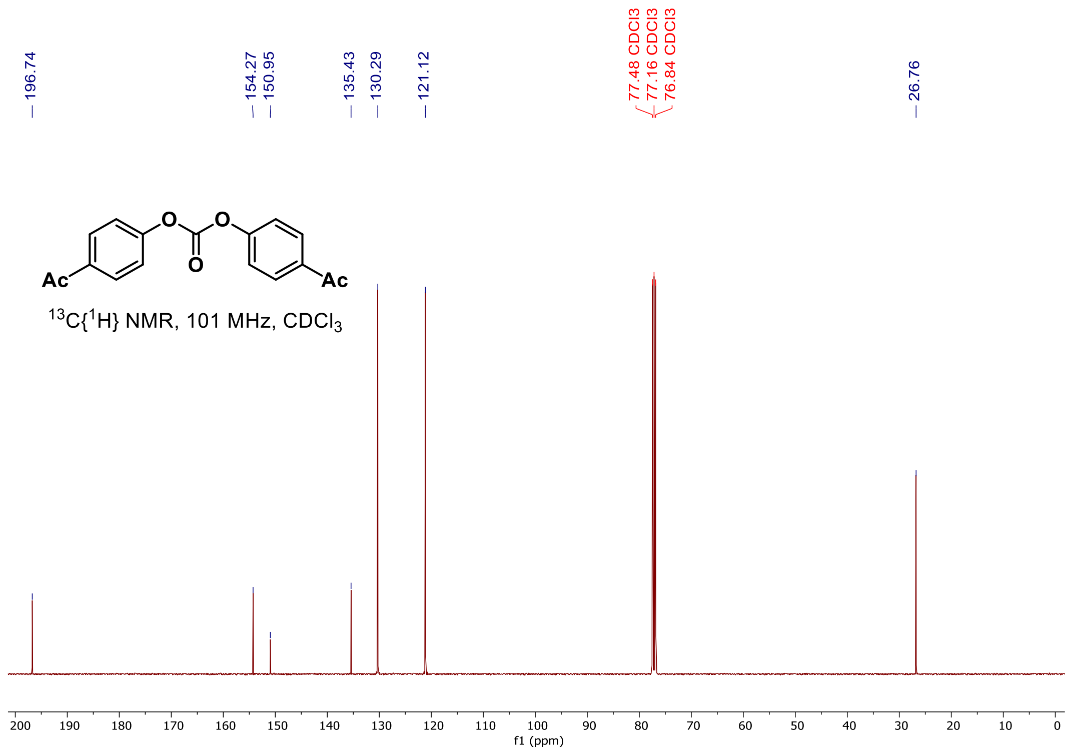

^{13}C NMR spectrum of compound **1b**

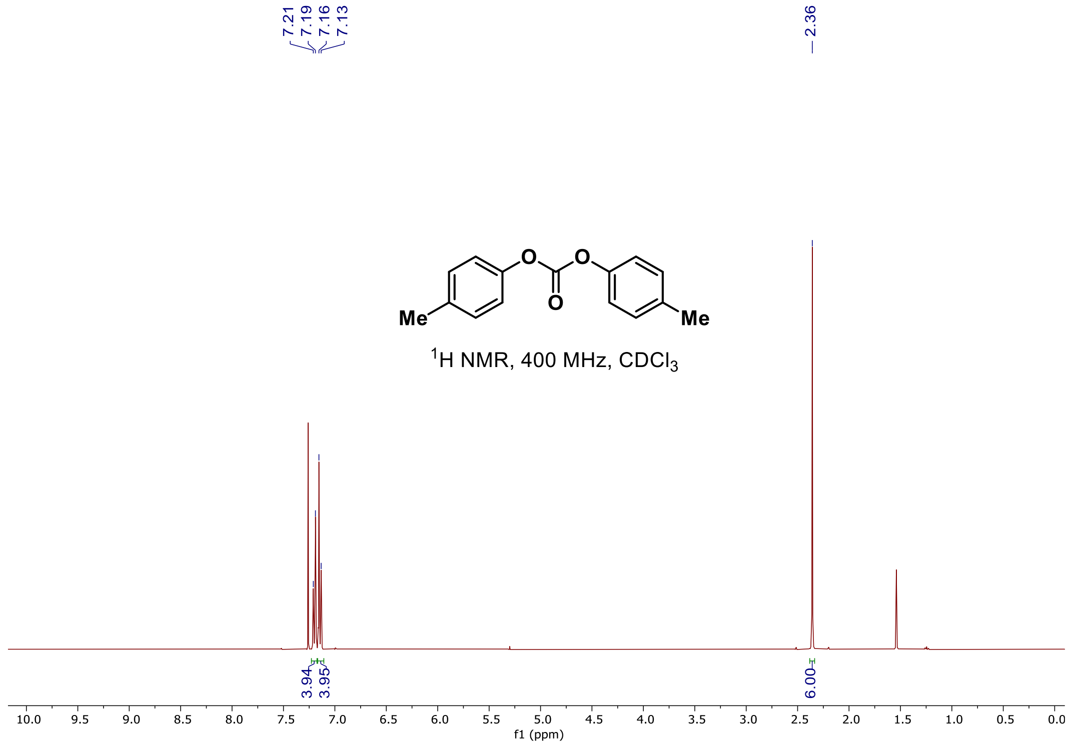

^{1}H NMR spectrum of compound **1c**

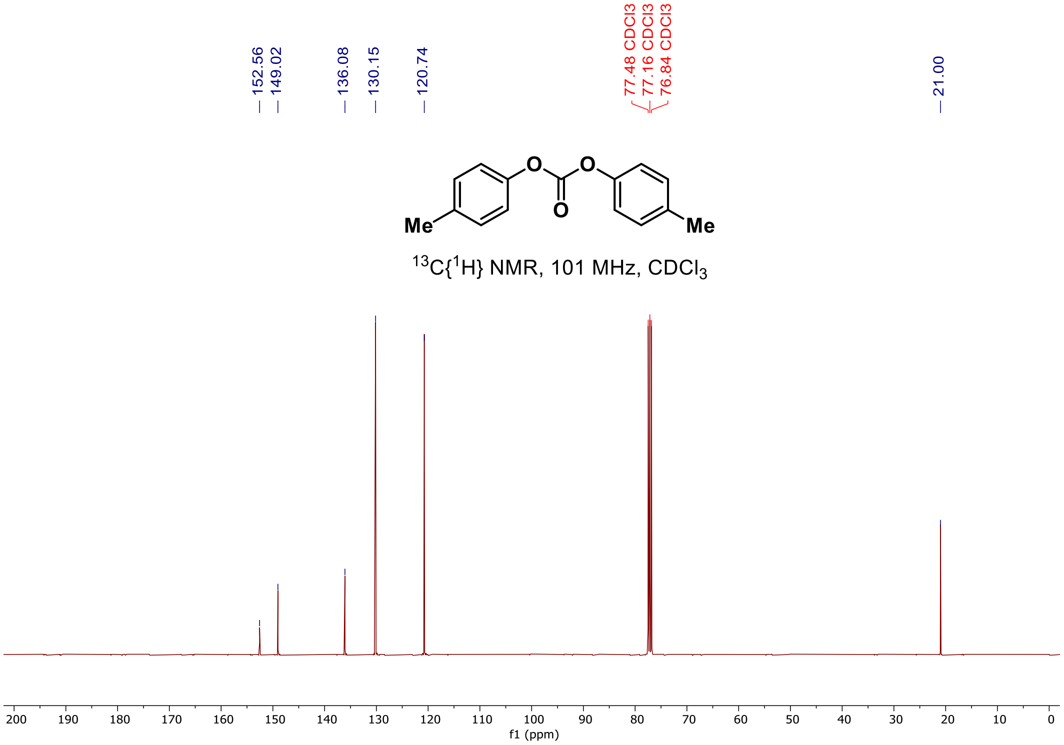

^{13}C NMR spectrum of compound **1c**

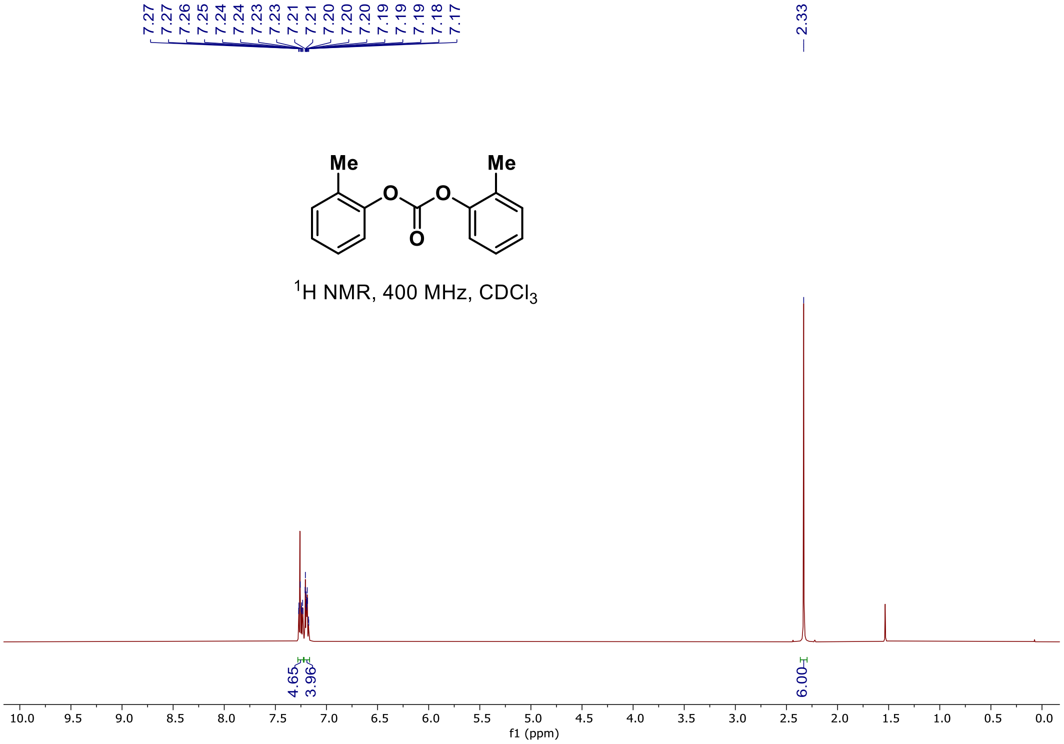

^{1}H NMR spectrum of compound **1d**

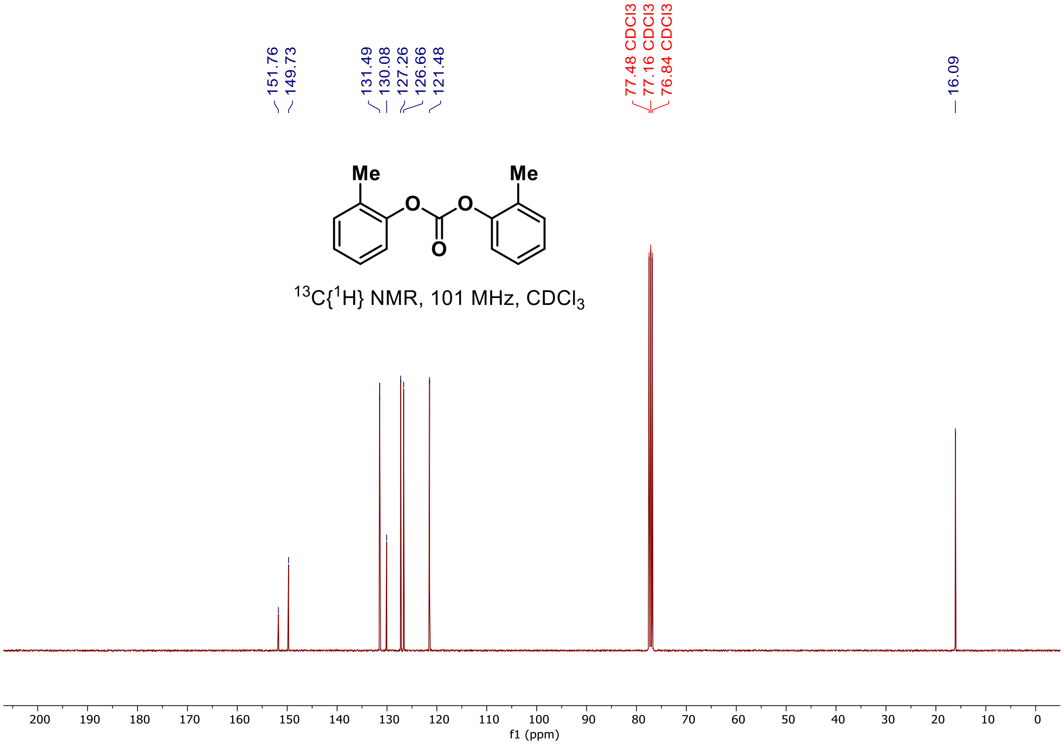

^{13}C NMR spectrum of compound **1d**

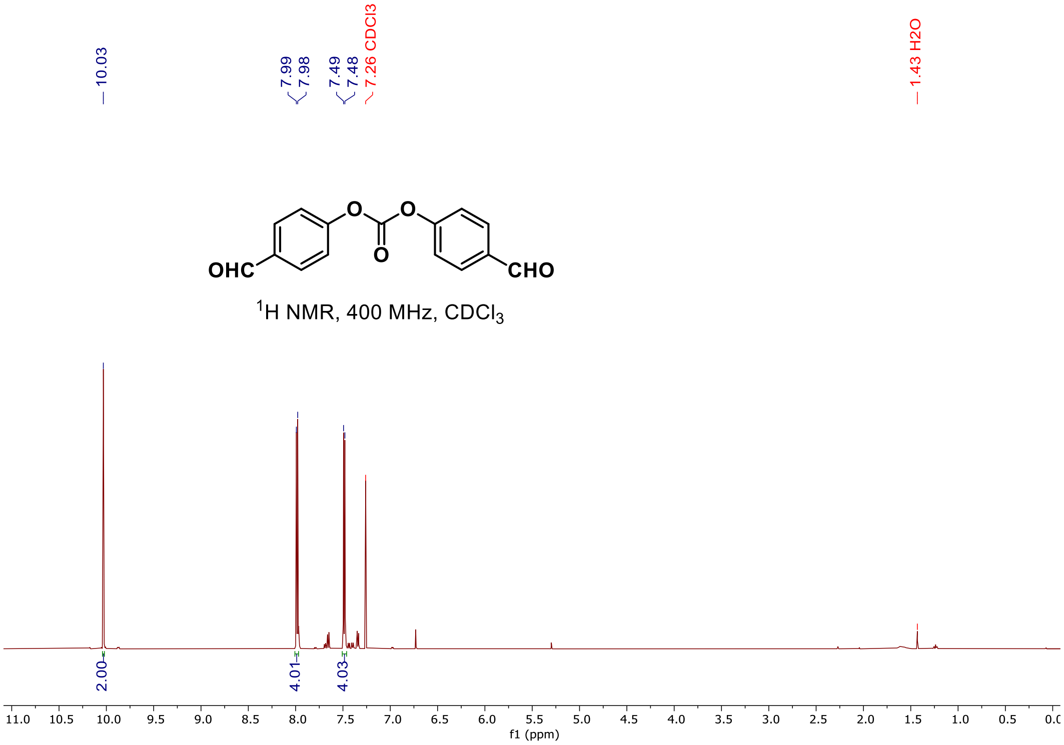

^{1}H NMR spectrum of compound **1f**

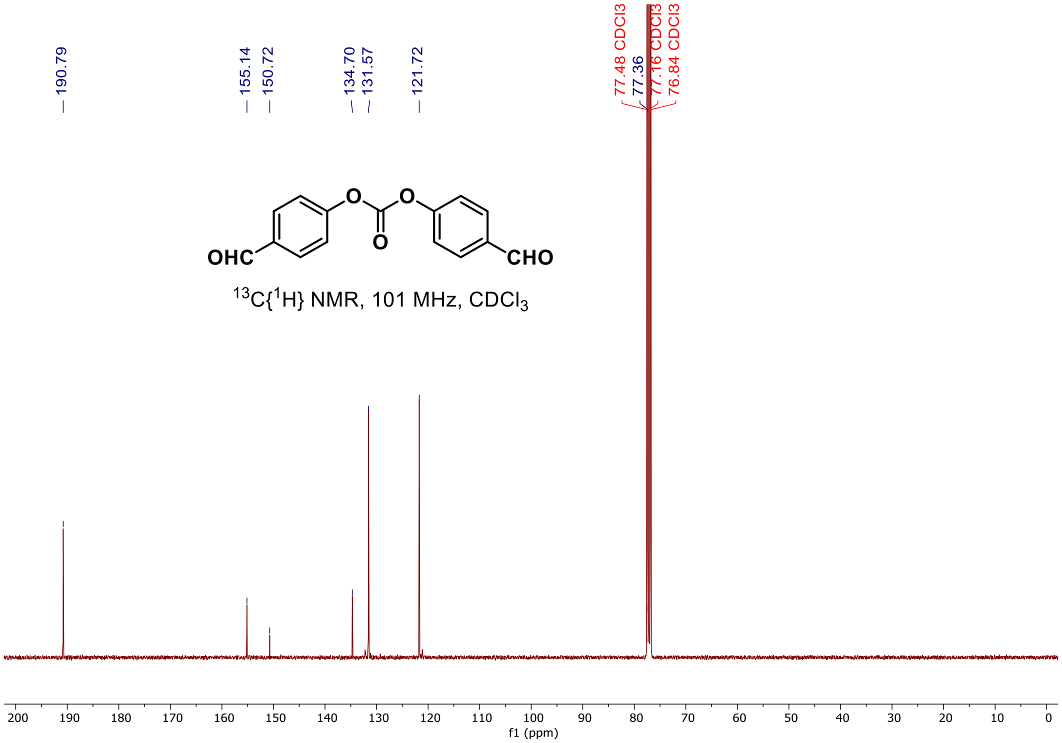

^{13}C NMR spectrum of compound **1f**

Mass spectra

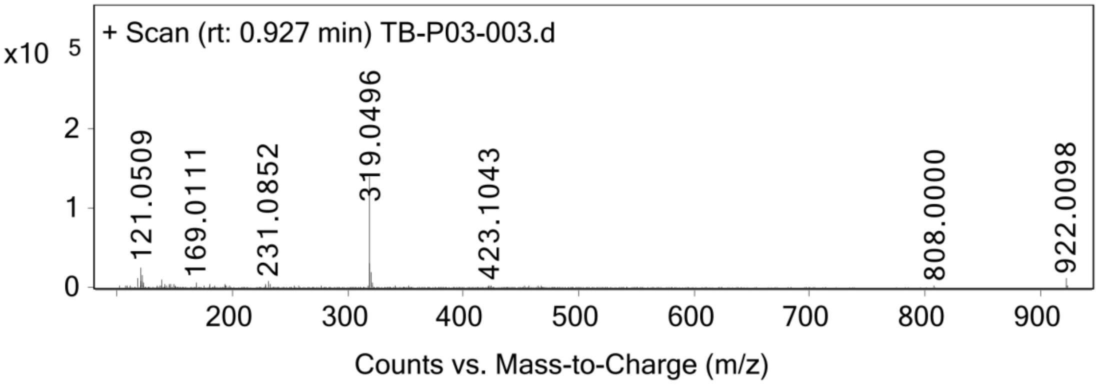

HRMS-ESI spectrum of compound **T**
